# Characteristics, Treatment, and Outcomes of Real-World Talazoparib-Treated Patients With Germline *BRCA*-Mutated Advanced HER2-Negative Breast Cancer

**DOI:** 10.1093/oncolo/oyad021

**Published:** 2023-03-23

**Authors:** Kristin M Zimmerman Savill, Jasmina Ivanova, Parisa Asgarisabet, Angelica Falkenstein, Alexandrina Balanean, Alexander Niyazov, Joanne C Ryan, Jonathan Kish, Ajeet Gajra, Reshma L Mahtani

**Affiliations:** Department of Real World Evidence and Insights, Cardinal Health Specialty Solutions, Cardinal Health, Dublin, OH, USA; Department of Patient Health & Impact, Pfizer Inc., New York, NY, USA; Department of Real World Evidence and Insights, Cardinal Health Specialty Solutions, Cardinal Health, Dublin, OH, USA; Department of Real World Evidence and Insights, Cardinal Health Specialty Solutions, Cardinal Health, Dublin, OH, USA; Department of Real World Evidence and Insights, Cardinal Health Specialty Solutions, Cardinal Health, Dublin, OH, USA; Department of Patient Health & Impact, Pfizer Inc., New York, NY, USA; Department of US Medical Affairs, Pfizer Inc., New York, NY, USA; Department of Real World Evidence and Insights, Cardinal Health Specialty Solutions, Cardinal Health, Dublin, OH, USA; Department of Real World Evidence and Insights, Cardinal Health Specialty Solutions, Cardinal Health, Dublin, OH, USA; Hematology-Oncology Associates of CNY, Syracuse, NY, USA; Miami Cancer Institute, Miami, FL, USA

**Keywords:** breast cancer, talazoparib, germline, *BRCA*, real-world

## Abstract

**Background:**

Talazoparib is a poly (adenosine diphosphate-ribose) polymerase inhibitor approved for the treatment of adult patients with deleterious or suspected deleterious germline *BRCA*-mutated (g*BRCA*m), HER2-negative, locally advanced or metastatic breast cancer (LA/mBC), with approval based on the EMBRACA trial. To date, there are no published data on talazoparib use in the real-world United States (USA) setting.

**Patients and Methods:**

Characteristics, treatment patterns, and clinical outcomes of real-world US patients with g*BRCA*m HER2-negative LA/mBC treated with talazoparib monotherapy were collected via retrospective chart review and summarized using descriptive statistics.

**Results:**

Among 84 eligible patients, 35.7% had hormone receptor-positive tumors and 64.3% had triple-negative LA/mBC (TNBC). At talazoparib initiation, 29.8% had ECOG PS of ≥2 and 19.0% had brain metastasis. Mutations in g*BRCA1* or *2* were detected among 64.3% and 35.7% of patients, respectively. Talazoparib was given as 1st-line therapy in 14.3% of patients, 2nd-line in 40.5%, and 3rd- or 4th-line in 45.2%. Median time to talazoparib treatment failure was 8.5 months (95% CI, 8.0-9.7), median progression-free survival was 8.7 months (95% CI, 8.0-9.9), the median time from initiation to chemotherapy was 12.2 months (95% CI, 10.5-20.1), and the overall response rate was 63.1%. No differences in clinical outcomes were observed between patients with HR-positive/HER2-negative LA/mBC and patients with TNBC by using unadjusted statistical comparisons. Brain metastasis and ECOG PS ≥2 at talazoparib initiation were associated with treatment failure and progression or mortality.

**Conclusion:**

Overall, talazoparib clinical outcomes in this real-world population are consistent with findings from EMBRACA.

## Teaser

Talazoparib was approved by the FDA for use in adult patients with germline BRCA mutated HER2-negative breast cancer based on results from the EMBRACA trial. This article reports the results of a study conducted in the real-world in a U.S. population.

**Implications for Practice**

This is the first study to describe patient characteristics, treatment patterns, and clinical outcomes among adult patients with germline *BRCA*-mutated, HER2-negative, locally advanced, or metastatic breast cancer treated with talazoparib in real-world US practice settings. The median time to talazoparib treatment discontinuation for any reason was 8.5 months and the talazoparib overall response rate was 63.1%. The median real-world progression-free survival for talazoparib was 8.7 months. The median time from initiation of talazoparib to chemotherapy was 12.2 months. Despite differences in baseline characteristics, real-world clinical outcomes were consistent with the EMBRACA trial, demonstrating the real-world clinical benefits of talazoparib treatment.

## Introduction

Breast cancer continues to be the leading cancer diagnosis and a significant contributor to cancer-related mortality with 290 560 new cases and 43 780 deaths estimated in the United States (USA) in 2022. The prognosis for locally advanced or metastatic breast cancer (LA/mBC) is poor, having an estimated 5-year survival rate of approximately 29.0% with distant metastasis.^1,2^ Treatment decision-making is typically informed by molecular characteristics including hormone receptor (HR) status, human epidermal growth factor receptor 2 (HER2) expression, and breast cancer susceptibility gene (*BRCA*) mutation status. Inherited, also known as germline, *BRCA* (g*BRCA*) mutations are associated with a higher lifetime risk of developing LA/mBC and experiencing poorer outcomes.^3^ However, these mutations are relatively rare, and are detected in less than 5% of unselected patients with metastatic disease.^4-7^ g*BRCA1/2* mutations are associated with worse breast cancer-specific survival compared with sporadic or negative *BRCA* status.^3^

Inhibition of poly (adenosine diphosphate–ribose) polymerase (PARP) activity has been demonstrated to effectively target tumors with deleterious mutations in *BRCA* by blocking the ability of cancer cells to effectively repair DNA damage.^7^ Since 2018, 2 PARP inhibitors (PARPis) have been approved by the US Food and Drug Administration (FDA) for the treatment of adult patients with deleterious or suspected deleterious g*BRCA* mutated (g*BRCA*m) HER2-negative advanced breast cancer: olaparib and talazoparib.^8,9^ Differences between these agents include PARP trapping potency and safety profiles.

Olaparib was approved in January, 2018 by the FDA for treatment of adult patients with g*BRCA*m HER2-negative metastatic breast cancer who received prior chemotherapy in the neoadjuvant, adjuvant, or metastatic setting, and, if the disease is HR-positive, had either received prior endocrine therapy or been considered inappropriate for it.^8^ Approval in this indication was based on phase III randomized trial, OlympiAD (NCT02000622), which demonstrated that olaparib was associated with superior progression-free survival (PFS) over physician’s choice of single-agent chemotherapy (capecitabine, vinorelbine, or eribulin) (7.0 vs. 4.2 months, respectively, hazard ratio 0.58; 95% CI, 0.43-0.80; *P* < .001).^6,7^ In addition, treatment with olaparib was associated with a higher objective response rate than treatment with chemotherapy (59.9% [95% CI, 52.0-67.4] vs. 28.8% [95% CI, 18.3-41.3], respectively).^6^

Talazoparib was approved by the FDA for use in adult patients with g*BRCA*m HER2-negative LA/mBC in October, 2018 based on the phase III EMBRACA trial (NCT01945775),^10^ which demonstrated that treatment with talazoparib in comparison to protocol-specific, non-platinum-based single-agent chemotherapy of the physician’s choice (ie, capecitabine, eribulin, gemcitabine, or vinorelbine) showed statistically significant improvement in PFS (median 8.6 vs. 5.6 months; hazard ratio 0.54; 95% CI, 0.41-0.71; *P* < .001]) and a significantly higher objective response rate (62.6% vs. 27.2%; odds ratio [OR] 5.0; 95% CI, 2.9-8.8; *P* < .001).^11,12^ The median treatment duration for EMBRACA was 6.9 months with talazoparib and 3.9 months with single-agent chemotherapy.^13^

Several studies have reported patient characteristics, treatment patterns, and outcomes for talazoparib-treated patients with advanced breast cancer in real-world settings in France,^14^ Turkey,^15^ and Russia.^16^ However, to the authors’ knowledge, there are no published studies to date on talazoparib utilization and outcomes in the real-world US setting. The objective of this retrospective chart review study (NCT04987931) was to describe the characteristics, treatment patterns, and clinical outcomes among adult patients with g*BRCA*m HER2-negative LA/mBC treated with talazoparib in real-world practice settings in the USA.

## Methods

### Data Source and Study Design

This study consisted of a retrospective, observational, physician-abstracted, and multi-site medical chart review. Medical oncologists from the Cardinal Health Oncology Provider Extended Network (OPEN) abstracted data from medical records of US patients selected based on prespecified eligibility criteria. OPEN is a geographically diverse, electronic medical record/group purchasing organization-agnostic, US community of >7000 oncologists, hematologists, and urologists in community practices ranging in size from private solo practitioners to hospital systems.

Study inclusion criteria required patients to have a diagnosis of g*BRCA*m HER2-negative LA/mBC to have initiated talazoparib monotherapy on or after October 16, 2018 (the date of FDA approval in this setting), to be at least 18 years of age at initiation, and to have at least 6 months of follow-up after initiation unless deceased within this time frame. Exclusion criteria included participation in any breast cancer clinical trial after initiation of talazoparib, treatment with a PARPi as neoadjuvant/adjuvant therapy, lack of known g*BRCA1/2* mutation, unknown HER2 status, and diagnosis with any other malignancy except carcinoma in situ or nonmelanoma skin cancer within the 5 years prior to data collection. Participating oncologists were instructed to select up to 20 eligible patients chronologically, starting with the earliest patient meeting the criteria, and selecting patients consecutively thereafter, to minimize selection bias. Physicians provided information about physician characteristics and abstracted data from medical records related to patient demographics, clinical characteristics, treatment patterns, and clinical outcomes (eg, response and progression) in an electronic case report form. Data were collected between August 20, 2021 and October 11, 2021.

The study received approval and exemption for obtaining patient informed consent by a central Institutional Review Board (IRB) (WCG IRB). The study followed accepted-standard research guidelines.

Clinical outcome measures for this study included time-to-treatment failure (TTF) for talazoparib, defined as the time from initiation of talazoparib to discontinuation for any reason, including disease progression, treatment toxicity, and death; real-world PFS (rwPFS) for talazoparib, defined as the time from initiation of talazoparib to charted disease progression or death from any cause, whichever occurred first; time from initiation of talazoparib to chemotherapy; real-world overall response rate (rwORR) for talazoparib, calculated as the sum of the number of physician-reported complete responses (CRs) and partial responses (PRs) divided by the total number of patients with reported disease response assessment; talazoparib real-world duration of response (rwDOR); and overall survival (OS) as the time from initiation of talazoparib to death from any cause.

For the evaluation of treatment patterns, a line of therapy for LA/mBC included chemotherapy-, hormonal therapy- and/or targeted therapy-based regimens. A line of therapy ended when the patient discontinued treatment with a regimen, added a new treatment to the regimen, or switched to a new regimen. The addition of a new treatment to an ongoing regimen was considered a new line of therapy. If a treatment used as part of combination therapy was held or discontinued, this was not considered a new line of therapy.

### Statistical Analysis

Data related to patient demographics and clinical characteristics, treatment patterns, and clinical outcomes were summarized using descriptive statistics. Analyses were conducted for the full final analysis cohort and 2 patient subgroups based on HR status (ie, patients with HR-positive disease and patients with triple-negative LA/mBC (TNBC). The Kaplan–Meier (KM) method was used to describe time-to-event outcomes for talazoparib including TTF, rwPFS, time from initiation of talazoparib to chemotherapy, rwDOR, and OS. Patients who were still alive or had not developed the progressive disease were censored on the date of talazoparib discontinuation or the last in-person or telemedicine visit at the physician’s practice, whichever occurred first. Statistical comparisons of clinical characteristics and outcomes between the HR-positive and TNBC subgroups were conducted using Chi-square tests for categorical variables and *t*-tests or Wilcoxon tests for continuous variables. Log-rank tests were used for subgroup comparisons of time-to-event outcomes. Cox proportional hazards (PH) regression was used to investigate the association of baseline demographic and clinical variables with TTF and rwPFS. All analyses were conducted in SAS v9.4.

## Results

### Patient Characteristics

In total, 84 patients treated by 9 community oncologists met eligibility criteria and were included in this analysis. Patient characteristics are shown in Table 1. Among the 84 patients, most were female (97.6%), White (71.4%), and non-Hispanic (84.5%). African American patients composed 16.7% of the study population, whereas 15.5% were of Hispanic ethnicity. Patients had a median age of 62 years at the initiation of talazoparib (minimum-maximum, 35.9-91.0) and geographically, were distributed across the USA (West, 57.1%; South, 20.2%; Midwest, 15.5%; and Northeast, 7.1%). At LA/mBC diagnosis, 50.0% of patients had commercial insurance, 41.7% had Medicare, and 11.9% had Medicaid.

**Table 1.** Patient demographic, clinical, and biomarker characteristics.

	All patients (*N* = 84)	HR+ (*n* = 30, 35.7% of total study population)	TNBC (*n* = 54, 64.3% of total study population)	*P*-value^a^
At diagnosis				
Age, years, median (min.-max.)	60.6 (33.8-91.0)	65.0 (47.2-91.0)	58.2 (33.8-78.3)	<.01
Sex, *n* (%)				.67
Female	82 (97.6)	29 (96.7)	53 (98.1)	
Male	2 (2.4)	1 (3.3)	1 (1.9)	
Race, *n* (%)				.09
White	60 (71.4)	22 (73.3)	38 (70.4)	
Black/African American	14 (16.7)	4 (13.3)	10 (18.5)	
Unknown^b^	5 (6.0)	0 (0.0)	5 (9.3)	
Asian	4 (4.8)	3 (10.0)	1 (1.9)	
Native Hawaiian or other Pacific Islander	1 (1.2)	1 (3.3)	0 (0.0)	
Ethnicity, *n* (%)				.76
Non-Hispanic/Latina/Latino	71 (84.5)	26 (86.7)	45 (83.3)	
Hispanic/Latina/Latino	13 (15.5)	4 (13.3)	9 (16.7)	
US region of residence, *n* (%)^c^				.09
West	48 (57.1)	13 (43.3)	35 (64.8)	
South	17 (20.2)	9 (30.0)	8 (14.8)	
Midwest	13 (15.5)	7 (23.3)	6 (11.1)	
Northeast	6 (7.1)	1 (3.3)	5 (9.3)	
Insurance at diagnosis with LA/mBC, *n* (%)^d^				
Commercial	42 (50.0)	6 (20.0)	36 (66.7)	<.01
Medicare	35 (41.7)	21 (70.0)	14 (25.9)	<.01
Medicaid	10 (11.9)	6 (20.0)	4 (7.4)	.16
At initiation of talazoparib				
Age, years, median (min.-max.)	62.0 (35.9-91.0)	68.6 (49.6-91.0)	59.1 (35.9-79.2)	<.01
AJCC stage IV, *n* (%)	84 (100.0)	30 (100.0)	54 (100.0)	NE
Visceral metastases, n (%)^e^	81 (96.4)	29 (96.7)	52 (96.3)	.93
Brain metastases, *n* (%)	16 (19.0)	3 (10.0)	13 (24.1)	.15
ECOG PS categorical score, *n* (%)^f^				.01
0/1	59 (70.2)	16 (53.3)	43 (79.6)	
2+	25 (29.8)	14 (46.7)	11 (20.4)	
g*BRCA* mutation, *n* (%)				.89
g*BRCA1*	54 (64.3)	19 (63.3)	35 (64.8)	
g*BRCA2*	30 (35.7)	11 (36.7)	19 (35.2)	
Panel type for g*BRCA* testing, *n* (%)				.24
Multigene panel	67 (79.8)	21 (70.0)	46 (85.2)	
Single-gene panel	4 (4.8)	2 (6.7)	2 (3.7)	
Unknown	13 (15.5)	7 (23.3)	6 (11.1)	
Timing of g*BRCA* testing relative to line of therapy, *n* (%)				.43
Prior to 1L	58 (69.0)	18 (60.0)	40 (74.1)	
During 1L	21 (25.0)	10 (33.3)	11 (20.4)	
Between 1L-2L	2 (2.4)	1 (3.3)	1 (1.9)	
During 3L	3 (3.6)	1 (3.3)	2 (3.7)	
Median (p25-p75) time from g*BRCA* testing to talazoparib initiation, months	15.8 (8.6-37.2)	28.9 (12.7-41.9)	13.7 (7.9-33.0)	.04
Other biomarkers, *n*/*n* tested (%)				
PD-L1 protein expression/mutations	21/44 (47.7)	0/0 (0.0)	21/44 (47.7)	NE
* PIK3CA* mutations	2/29 (6.9)	2/17 (11.8)	0/12 (0.0)	.49
* ESR1* mutations	1/8 (12.5)	1/2 (50.0)	6/6 (100.0)	.25

^a^Statistical comparisons between HR-positive and TNBC subgroups.

^b^Race was entered as Hispanic, Latino, or Latina via open text. However, the US census considers Hispanic/Latino/Latina as ethnicity rather than race, and therefore race is unknown.

^c^Northeast includes Connecticut, Delaware, Massachusetts, Maine, Maryland, New Hampshire, New Jersey, New York, Pennsylvania, Rhode Island, Vermont; Midwest includes Iowa, Illinois, Indiana, Kansas, Michigan, Minnesota, Missouri, North Dakota, Nebraska, Ohio, South Dakota, Wisconsin; South includes Arkansas, Alabama, District of Columbia, Georgia, Florida, Kentucky, Louisiana, Mississippi, North Carolina, Oklahoma, South Carolina, Tennessee, Texas, Virginia, West Virginia; West includes Alaska, Arizona, California, Colorado, Hawaii, Idaho, Montana, New Mexico, Nevada, Oregon, Utah, Washington, Wyoming.

^d^Not mutually exclusive.

^e^Visceral metastases defined as metastases in the adrenal gland; gastrointestinal system; genitourinary system; ovary, gynecologic system; liver; lung; pleura, pericardial, and/or peritoneal cavity at the time of initiation of talazoparib.

^f^ECOG PS scoring definitions:

0—Fully active; no restriction

1—Restricted in strenuous physical activities; fully ambulatory and able to carry out light work.

2—Capable of all self-care but unable to carry out any work activities; up and about >50% of waking hours.

3—Capable of only limited self-care; confined to bed or chair >50% of waking hours.

4—Completely disabled; could not carry out any self-care; totally confined to bed or chair.

Abbreviations: AJCC, American Joint Committee on Cancer; ECOG PS, Eastern Cooperative Oncology Group Performance Status; ESR1, estrogen receptor alpha; g*BRCA*m, germline breast cancer susceptibility gene-mutated; gBRCA1, germline breast cancer susceptibility gene 1; gBRCA2, germline breast cancer susceptibility gene 2; HER2-, human epidermal growth factor receptor 2-negative; HR+, hormone receptor-positive; 1L, 1st-line; 2L, 2nd-line; 3L, 3rd-line; LA/mBC, locally advanced or metastatic breast cancer; max, maximum; min, minimum; mBC, advanced/metastatic breast cancer; NE, could not be estimated; p25, 25th percentile; p75, 75th percentile; PD-L1, programmed death ligand 1; PIK3CA, phosphatidylinositol-4,5-bisphosphate 3-kinase, catalytic subunit alpha; SD, standard deviation; TNBC, triple-negative breast cancer.

At the initiation of talazoparib, all patients had stage IV disease, 96.4% had visceral metastases, and 19.0% had brain metastases. Approximately 30.0% had an Eastern Cooperative Oncology Group performance status (ECOG PS) ≥2 at the initiation. A g*BRCA1* mutation was detected in 64.3% of patients, whereas a g*BRCA2* mutation was detected in 35.7% of patients. g*BRCA1/2* mutations were identified from testing using multigene panels among 79.8% of patients, single gene testing among 4.8%, and unknown panel types among 15.5%. Testing most frequently occurred prior to 1st-line (1L) therapy for LA/mBC (69.0% of patients). Testing for g*BRCA1/2* was conducted during 1L treatment for metastatic disease in 25.0% of patients, between 1L and second-line (2L) in 2.4%, and during third-line (3L) in 3.6% of patients. Median (25th percentile to 75th percentile [p. 25-75]) time to talazoparib initiation after g*BRCA* mutation testing results was 15.8 months (8.6-37.2 months). Among 44 patients with triple-negative LA/mBC (TNBC) and programmed death ligand 1 (PD-L1) testing results available, PD-L1 positivity was noted in 47.7% of patients. In addition, among patients with available testing results, *PIK3CA* mutations were found in 2 of 29 patients (6.9%) and *ESR1* mutations were found in 1 of 8 patients (12.5%).

HR-positivity was reported for 35.7% (*n* = 30) of patients and TNBC classification was reported for 64.3% (*n* = 54). Although similar distributions across sex, race, and ethnicity were observed between patients with HR-positive disease and those with TNBC, the former had a higher median age at initiation of talazoparib (68.6 vs. 59.1 years, *P* < .01). Differences in geographic distribution were observed between these subgroups, with a higher proportion of patients with TNBC from the West. Furthermore, a lower proportion of patients with HR-positive disease had commercial insurance than did patients with TNBC (20.0% vs. 66.7%, *P* < .01), and a higher proportion of patients with HR-positive disease were covered by Medicare than were patients with TNBC (70.0% vs. 25.9%, *P* < .01). A significantly higher proportion of patients with HR-positive disease had an ECOG PS ≥2 at baseline as compared to patients with TNBC (46.7% vs. 20.4%, respectively, *P* = .01). No statistically significant differences were identified between HR-positive and TNBC subgroups in terms of proportions with visceral metastases or brain metastases.

### Treatment Patterns

Patient treatment patterns are shown in Supplementary Fig. S1; Table 2. Among all 84 patients, talazoparib was prescribed as 1L therapy for 14.3%, 2L for 40.5%, 3L for 34.5%, and 4L for 10.7%. A greater proportion of patients with TNBC received talazoparib as 1L than did those with HR-positive disease (20.4% vs. 3.3%) and as 2L (46.3% vs. 30.0%). Whereas talazoparib was most frequently given as 2L therapy among patients with TNBC, it was most often administered as 3L therapy among those with HR-positive disease (Table 2).

**Table 2.** Patient treatment patterns.

	All patients (*N* = 84)	HR+ (*n* = 30)	TNBC (*n* = 54)
Line of therapy that talazoparib was administered for the treatment of LA/mBC, *n* (%)			
1^st^	12 (14.3)	1 (3.3)	11 (20.4)
2^nd^	34 (40.5)	9 (30.0)	25 (46.3)
3^rd^	29 (34.5)	13 (43.3)	16 (29.6)
4^th^	9 (10.7)	7 (23.3)	2 (3.7)
Dosage of talazoparib at initiation, *n* (%)			
1 mg daily	77 (91.7)	25 (83.3)	52 (96.3)
0.75 mg daily	7 (8.3)	5 (16.7)	2 (3.7)
Talazoparib dosage modification (reduction or temporary interruption), *n* (%)^a^	13 (15.5)	8 (26.7)	5 (9.3)
Reduction, *n* (%)	12 (92.3)	8 (100.0)	4 (80.0)
Median (p25-p75) time to first reduction (weeks)	8.9 (4.2-12.9)	8.9 (5.5-10.9)	9.3 (4.1-14.4)
Temporary interruption, *n* (%)	5 (38.5)	2 (25.0)	3 (60.0)
Median (p25-p75) time to first interruption (weeks)	9.0 (7.9-9.6)	10.9 (4.6-17.3)	9.0 (7.9-9.6)
Supportive therapy received during talazoparib treatment, *n* (%)^a^			
Yes	63 (75.0)	21 (70.0)	42 (77.8)
Antidiarrheal	47 (56.0)	15 (50.0)	32 (59.3)
Antiemetics	54 (64.3)	16 (53.3)	38 (70.4)
Bone stimulating agents	14 (16.7)	1 (3.3)	13 (24.1)
ESAs (erythropoiesis-stimulating agents)	1 (1.2)	1 (3.3)	0 (0.0)
G-CSF (granulocyte-colony stimulating factor)	1 (1.2)	0 (0.0)	1 (1.9)
IV fluids	2 (2.4)	1 (3.3)	1 (1.9)
Platelet stimulating agents	0 (0.0)	0 (0.0)	0 (0.0)
Steroids	4 (4.8)	3 (10.0)	1 (1.9)
Packed red blood cells Mean (SD) transfusions per patient receiving them	7 (8.3) 2.1 (1.1)	4 (13.3) 2.5 (1.3)	3 (5.6) 1.7 (0.6)
Platelet transfusion(s)	0 (0.0)	0 (0.0)	0 (0.0)
No	21 (25.0)	9 (30.0)	12 (22.2)
(Neo)adjuvant chemotherapy and/or hormonal therapy prior to talazoparib, *n* (%)^a^	33 (39.3)	9 (30.0)	24 (44.4)
Systemic therapy received for LA/mBC prior to talazoparib, *n* (%)	72 (85.7)	29 (96.7)	43 (79.6)
Treatment regimens received as any line of therapy prior to talazoparib, among patients with prior treatment) *n* (%)^a^			
Single-agent HT	8 (11.1)	8 (27.6)	0 (0.0)
Combo HT	7 (9.7)	7 (24.1)	0 (0.0)
Single-agent CT (NPt-based)	26 (36.1)	9 (31.0)	17 (39.5)
Combo CT (Pt-based)	18 (25.0)	0 (0.0)	18 (41.9)
Combo CT (NPt-based)	13 (18.1)	2 (6.9)	11 (25.6)
Combo CDK4/6i + HT	26 (36.1)	26 (89.7)	0 (0.0)
Combo mTORi + HT	3 (4.2)	3 (10.3)	0 (0.0)
Combo I-O + combo CT (Pt-based)	1 (1.4)	0 (0.0)	1 (2.3)
Combo I-O + CT (NPt-based)	12 (16.7)	0 (0.0)	12 (27.9)
Combo *PI3KI* + HT	1 (1.4)	1 (3.4)	0 (0.0)
Chemotherapy received for LA/mBC post-talazoparib, *n* (%)	21 (25.0)	6 (20.0)	15 (27.7)

^a^Not mutually exclusive.

Abbreviations: CDK4/6i, cyclin dependent kinase 4/6 inhibitor; combo, combination; CT, chemotherapy; g*BRCA*m, germline breast cancer susceptibility gene mutated; HER2, human epidermal growth factor receptor; HER2-, human epidermal growth factor receptor 2 negative; HR+, hormone receptor-positive; HT, hormonal therapy; I-O, immuno-oncology therapy; LA/mBC, locally advanced or metastatic breast cancer; mTORi, mammalian target of rapamycin inhibitor; (neo)adjuvant, neoadjuvant or adjuvant; NPt, non-platinum; p25, 25th percentile; p75, 75th percentile; PI3Ki, phosphatidylinositol-3-kinase inhibitor; Pt, platinum; TNBC, triple-negative breast cancer.

Most patients (91.7%) initiated talazoparib at a dosage of 1 mg daily, and the remainder (8.3%) at 0.75 mg daily (Table 2). Overall, 15.5% of patients required a dosage reduction or temporary interruption during talazoparib therapy. Supportive care, including medications prescribed as needed or for prophylactic use (the distinction was not made for this study), was administered in 75.0% of patients. The most prescribed supportive care agents were antiemetics (64.3% of patients) and antidiarrheal agents (56.0% of patients). Approximately 8.3% of patients received packed red blood cell transfusions (mean [standard deviation (SD)] transfusions per patient, 2.1 [1.1]) (Table 2).

Overall, in the early-stage setting, 39.3% of patients received (neo)adjuvant chemotherapy and/or hormonal therapy and 85.7% received systemic therapy (which may have included chemotherapy, hormonal therapy, targeted therapy, and/or immune therapy) for LA/mBC prior to receiving talazoparib (Table 2). The most common regimens received for LA/mBC prior to talazoparib in HR-positive patients were combination cyclin-dependent kinase 4/6 (CDK4/6)-targeted therapy and hormonal therapy (89.7%), single-agent non-platinum-based chemotherapy (31.0 %), single-agent hormonal therapy (27.6%), and combination hormonal therapy (24.1%). The most common regimens received prior to talazoparib in patients with TNBC were combination platinum-based chemotherapy (41.9%), single-agent non-platinum-based chemotherapy (39.5%), combination immuno-oncology therapy (I-O) and non-platinum-based chemotherapy (27.9%), and combination non-platinum-based chemotherapy (25.6%). Following talazoparib therapy, 23.3% of patients with HR-positive disease received systemic therapy and 20.0% specifically received a chemotherapy-based regimen post-talazoparib (Table 2; Supplementary Table S1). Among patients with TNBC, 29.6% of patients received systemic therapy and 27.8% specifically received chemotherapy-based treatment following talazoparib (Table 2; Supplementary Table S1).

### Clinical Outcomes

**Figure 1.**
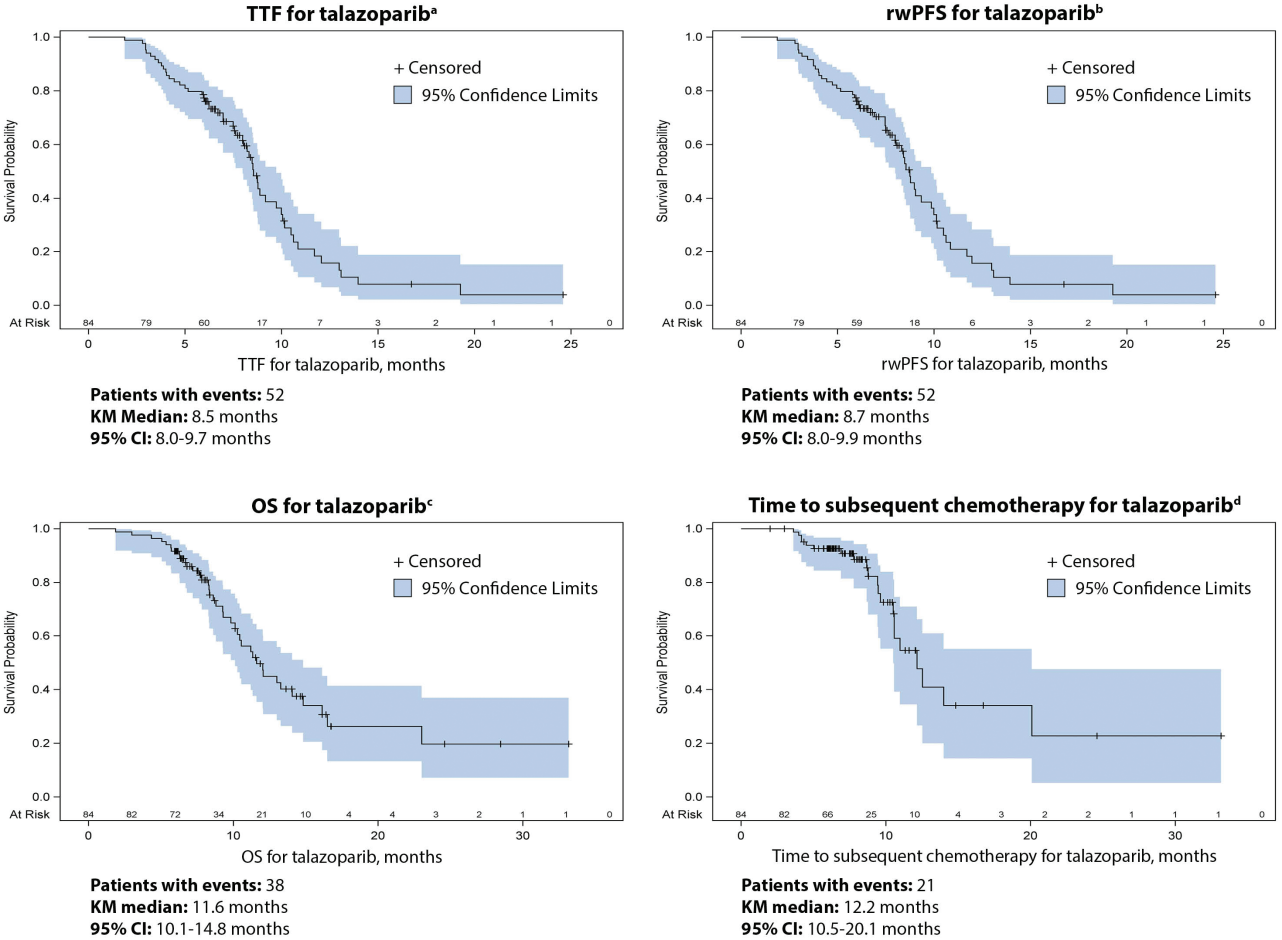
KM curves for talazoparib TTF, rwPFS, OS, and time to subsequent chemotherapy. ^a^Time from talazoparib initiation to discontinuation for any reason. Patients still on therapy at last encounter were censored at last encounter date. ^b^Time from initiation of talazoparib to charted disease progression based on radiographic imaging or death from any cause, whichever occurred first. Patients who discontinued talazoparib for a reason other than progression or death were censored at talazoparib discontinuation date. Patients still receiving talazoparib at last encounter were censored on date of last encounter. ^c^Time from initiation of talazoparib to death. Patients who were known to be alive or lost to follow-up were censored on the date of last encounter. ^d^Time from initiation of talazoparib to initiation of subsequent chemotherapy, received as any line of therapy after talazoparib. Patients who had not received chemotherapy at last encounter were censored at last encounter date or date of death, whichever occurred first. Abbreviations: CI, confidence interval; g*BRCA*m, germline breast cancer susceptibility gene mutated; HER2–, human epidermal growth factor receptor 2 negative; KM, Kaplan-Meier; LA/mBC, locally advanced or metastatic breast cancer; OS, overall survival; rwPFS, real-world progression-free survival; TTF, time-to-treatment failure.

Clinical outcomes are shown in Table 3; Fig. 1. The median duration of follow-up after talazoparib initiation was 8.2 months (minimum-maximum, 2.0-33.2 months) (Table 3). Among all patients, the median TTF of talazoparib was 8.5 months (95% CI, 8.0-9.7) (Table 3, Fig. 1). The median rwPFS for talazoparib was 8.7 months (95% CI, 8.0-9.9) (Table 3, Fig. 1). The median time from talazoparib initiation to subsequent chemotherapy initiation (received as any line of therapy after talazoparib) was 12.2 months (95% CI, 10.5-20.1 months) (Table 3, Fig. 1). In addition, the median OS for talazoparib was 11.6 months (95% CI, 10.1-14.8 months), although the rate of censoring was >50% due to the fact that the majority of patients (54.8%) were alive at data collection (Table 3; Fig. 1). The rwORR for talazoparib was 63.1% (95% CI, 52.2-74.0), and the median DOR was 7.1 months (95% CI, 5.5-8.7 months) (Table 3).

**Table 3.** Clinical outcomes.

	All patients (*N* = 84)	HR+ (*n* = 30)	TNBC (*n* = 54)	*P*-value^a^
Median (min.-max.) duration of follow-up since talazoparib initiation, months	8.2 (2.0-33.2)	9.0 (6.0-28.5)	7.8 (2.0-33.2)	.15
TTF for talazoparib^b^				
KM median (95% CI), months	8.5 (8.0-9.7)	8.5 (8.0-10.6)	8.8 (7.0-10.1)	.51
*n* of events	52	19	33	
rwPFS for talazoparib^c^				
KM median (95% CI), months	8.7 (8.0-9.9)	8.5 (8.0-10.6)	9.0 (7.5-10.1)	.56
*n* of events	52	19	33	
Best response to talazoparib, *n* (%)				
CR	3 (3.6)	0 (0.0)	3 (5.6)	.45
PR	50 (59.5)	21 (70.0)	29 (53.7)	
Stable disease	19 (22.6)	5 (16.7)	14 (25.9)	
Progressive disease	10 (11.9)	4 (13.3)	6 (11.1)	
Not evaluable	2 (2.4)	0 (0.0)	2 (3.7)	
rwORR to talazoparib^d^				
*n*	53	21	32	
% (95% CI)	63.1 (52.2-74.0)	70.0 (51.9-88.1)	59.3 (45.2-73.3)	.33
rwDOR to talazoparib^e^				
KM median (95% CI), months	7.1 (5.5-8.7)	7.5 (5.0-10.1)	7.0 (5.4-8.1)	.46
*n* of events	22	9	13	
Time from initiation of talazoparib to chemotherapy^f^				
KM median (95% CI), months	12.2 (10.5-20.1)	20.1 (9.6-NE)	11.0 (9.5-14.0)	.19
*n* of events	21	6	15	
Alive at data collection, *n* (%)	46 (54.8)	16 (53.3)	30 (55.6)	.84
OS for talazoparib^g^				
KM median (95% CI), months	11.6 (10.1-14.8)	12.0 (10.3-13.3)	11.6 (8.6-16.5)	.72
*n* of events	38	14	24	

^a^Statistical comparisons between HR-positive/HER2- and TNBC subgroups. Comparisons between subgroups did not adjust for differences in patient characteristics.

^b^Time from talazoparib initiation to discontinuation for any reason. Patients still on therapy at last encounter were censored at last encounter date.

^c^Time from initiation of talazoparib to charted disease progression based on radiographic imaging or death from any cause, whichever occurred first. Patients who discontinued talazoparib for a reason other than progression or death were censored at talazoparib discontinuation date. Patients still receiving talazoparib at last encounter were censored on date of last encounter.

^d^Sum of complete and partial responses reported as best response divided by all patients with reported disease response assessment.

^e^Time from initial documentation of disease response to talazoparib (complete or partial) to disease progression or death. Patients who did not progress and were reported to be alive or lost to follow-up were censored at last encounter date.

^f^Time from initiation of talazoparib to initiation of subsequent chemotherapy, received as any line of therapy after talazoparib. Patients who had not received chemotherapy at last encounter were censored at last encounter date or date of death, whichever occurred first.

^g^Time from initiation of talazoparib to death. Patients who were known to be alive or lost to follow-up were censored on the date of last encounter.

Abbreviations: CI, confidence interval; CR, complete response; g*BRCA*m, germline breast cancer susceptibility gene mutated; HER2-, human epidermal growth factor receptor 2 negative; HR+, hormone receptor-positive; KM, Kaplan–Meier; LA/mBC, locally advanced or metastatic breast cancer; max., maximum; min., minimum; OS, overall survival; PR, partial response; rwDOR, real-world duration of response; rwORR, real-world overall response rate; rwPFS, real-world progression-free survival; TNBC, triple-negative breast cancer; TTF, time-to-treatment failure.

In unadjusted comparisons, no statistically significant differences were detected between patients with HR-positive disease and patients with TNBC in talazoparib TTF, rwPFS, time from talazoparib initiation to subsequent chemotherapy initiation, rwORR, or rwDOR (Table 3; Fig. 1).

Patient characteristics associated with TTF and rwPFS were identified using univariate and multivariate analyses, the latter of which adjusted for age at initiation of talazoparib, HR status, race, brain metastasis at the initiation of talazoparib, ECOG PS at the initiation of talazoparib, and line of therapy for LA/mBC in which talazoparib was received. In univariate and multivariate Cox PH model analyses, the presence of brain metastases and ECOG PS of ≥2 was associated with a significantly higher risk of treatment failure (respective TTF hazard ratios, 2.3; 95% CI, 1.1-4.8; *P* = .02 and 2.7; 95% CI, 1.3-5.8; *P* = .01) and significantly higher risk of progression or death (respective rwPFS hazard ratios, 2.4; 95% CI, 1.2-5.0; *P* = .02 and 2.7; 95% CI, 1.3-5.8; *P* = .01) (Table 4).

**Table 4.** Cox proportional hazards for characteristics associated with talazoparib TTF and rwPFS.

	Univariate hazard ratio (95% CI)	*P*-value^a^	Multivariate^b^ hazard ratio (95% CI)	*P*-value^c^
TTF				
Age at initiation of talazoparib ≥50 years (reference: <50 years)	1.0 (0.5-1.9)	.91	0.9 (0.4-1.8)	.69
≥12 months from initial diagnosis of BC to diagnosis of LA/mBC among patients with stage Ia, Ib, IIa, or stage IIb at initial diagnosis (reference: <12 months)	0.6 (0.2-1.7)	.34	—	—
Non-White race (reference: White)	0.7 (0.4-1.3)	.24	0.9 (0.5-1.8)	.84
LA/mBC molecular subtype TNBC (reference: HR-positive)	1.2 (0.7-2.2)	.51	1.5 (0.7-3.1)	.29
Brain metastasis at initiation of talazoparib (reference: no)	3.2 (1.7-5.8)	<.01	2.3 (1.1-4.8)	.02
ECOG PS 2+ at initiation of talazoparib^d^ (reference: 0-1)	2.6 (1.5-4.6)	<.01	2.7 (1.3-5.8)	.01
g*BRCA2* mutation (reference: g*BRCA1* mutation)	1.4 (0.8-2.5)	.22	—	—
Talazoparib received in 1L/2L (reference: 3L+)	0.6 (0.3-1.0)	.05^e^	1.0 (0.4-2.0)	.90
Platinum-based therapy received prior to talazoparib^f^ (reference: no)	1.4 (0.7-2.8)	.27	—	—
Insurance at diagnosis with LA/mBC^g^				
Medicaid (reference: Medicare)	2.0 (0.8-5.0)	.12	—	—
Commercial (reference: Medicare)	0.8 (0.4-1.5)	.46	—	—
rwPFS				
Age at initiation of talazoparib ≥50 years (reference: <50 years)	0.9 (0.5-1.8)	0.83	0.8 (0.4-1.7)	.62
≥12 months from initial diagnosis of BC to diagnosis of LA/mBC among patients with Stage Ia, Ib, IIa, or Stage IIb at initial diagnosis (reference: <12 months)	0.7 (0.2-2.0)	0.50	—	—
Non-White race (reference: White)	0.7 (0.4-1.2)	0.20	0.9 (0.5-1.7)	.74
LA/mBC molecular subtype TNBC (reference: HR-positive)	1.2 (0.7-2.1)	0.56	1.4 (0.7-3.0)	.34
Brain metastasis at initiation of talazoparib (reference: no)	3.2 (1.7-5.9)	<.01	2.4 (1.2-5.0)	.02
ECOG PS 2+ at initiation of talazoparib^d^ (reference: 0-1)	2.7 (1.5-4.6)	<.01	2.7 (1.3-5.8)	<.01
g*BRCA2* mutation (reference: g*BRCA1* mutation)	1.4 (0.8-2.5)	.23	—	—
Talazoparib received in 1L/2L (reference: 3L+)	0.6 (0.3-1.0)	.04	1.0 (0.5-2.1)	.94
Platinum-based therapy prior to talazoparib^f^ (reference: no)	1.4 (0.7-2.8)	.29	—	—
Insurance at diagnosis with LA/mBC^g^				
Medicaid (reference: Medicare)	2.5 (1.0-6.3)	.06	—	—
Commercial (reference: Medicare)	0.8 (0.4-1.7)	.65	—	—

^a^ *P*-value from univariate analysis.

^b^Multivariate analysis adjusted for age at initiation with talazoparib, hormone receptor status, race, brain metastases at initiation of talazoparib, ECOG PS at initiation of talazoparib, and line of therapy for LA/mBC in which talazoparib was received.

^c^ *P*-value from multivariate analysis.

^d^ECOG PS = Eastern Cooperative Oncology Group performance status. Scoring definitions:

0—Fully active; no restriction.

1—Restricted in strenuous physical activities; fully ambulatory and able to carry out light work.

2—Capable of all self-care but unable to carry out any work activities; up and about >50% of waking hours.

3—Capable of only limited self-care; confined to bed or chair >50% of waking hours.

4—Completely disabled; could not carry out any self-care; totally confined to bed or chair.

^e^ *P*-value was 0.0468, and therefore below the statistical significance threshold of 0.05.

^f^Includes platinum-based therapy received as adjuvant or neoadjuvant therapy or as therapy for LA/mBC received prior to talazoparib.

^g^For patients who had Medicare and commercial insurance at diagnosis, insurance was categorized as “commercial insurance” and for patients who had Medicare and Medicaid at diagnosis, insurance was categorized as “Medicaid.”

Abbreviations: CI, confidence interval; ECOG PS, Eastern Cooperative Oncology Group performance status; gBRCA1, germline breast cancer susceptibility gene 1; gBRCA2, germline breast cancer susceptibility gene 2; g*BRCA*m, germline breast cancer susceptibility gene mutated; HER2-negative, human epidermal growth factor receptor 2 negative; 1L, 1st-line; 2L, 2nd-line; LA/mBC, locally advanced or metastatic breast cancer; rwPFS, real-world progression-free survival; TNBC, triple-negative breast cancer; TTF, time to treatment failure.

## Discussion

This study aimed to describe talazoparib utilization and clinical outcomes in the initial 3 years post-FDA approval among US adult patients with g*BRCA*m HER2-negative LA/mBC. This study was the first real-world evaluation conducted in the USA. However, similar studies have been conducted in France,^14^ Turkey,^15^ and Russia^16^ and have been published.

Patient demographic and clinical characteristics differed between this real-world study and the EMBRACA trial. Patients in this real-world study were older (median age at talazoparib initiation of 62 vs. 45 years in EMBRACA), had worse performance status (ECOG PS at talazoparib initiation of 2+ among 29.8% vs. 2.1% in EMBRACA), and were treated with talazoparib in later lines of therapy (talazoparib was received as 3L or 4L therapy among 45.2% of patients in this study, whereas in EMBRACA, 24.0% of patients initiated talazoparib after 2 or 3 prior cytotoxic regimens for advanced breast cancer).^11,13^ Despite these differences, median rwPFS and rwORR for talazoparib observed for this real-world US cohort were consistent with those reported for patients in the talazoparib arm of the EMBRACA trial. The median rwPFS for talazoparib among real-world US patients was 8.7 months (median follow-up 8.2 months), and the rwORR was 63.1%; for patients in EMBRACA, the median PFS for talazoparib was 8.6 months (median follow-up 11.2 months) and the objective response rate was 62.6%.^10-13^ The median OS of 11.6 months from talazoparib initiation observed in this real-world study was shorter than the median OS of 19.3 months observed in the EMBRACA trial.^13^ However, in this real-world study, the median duration of follow-up was only 8.2 months, the rate of censoring was >50%, and patients were older, with worse performance status, and more heavily pretreated than in EMBRACA. Notable differences between this real-world study and EMBRACA were observed in transfusions received during talazoparib treatment and in talazoparib dosage reductions or interruptions. Among talazoparib-treated patients in the EMBRACA trial, 39.2% received at least 1 red blood cell transfusion and 3.5% received platelet transfusion; the rates in this study were 8.3% and 0.0%, respectively.^13^ Talazoparib dosage reductions and interruptions due to adverse events in the EMBRACA trial were reported in 53.1% and 62.6% of patients, respectively. In this real-world study, 14.3% of patients had talazoparib dosage reduction for any reason and 6.0% had temporary dosage interruption for any reason. These differences may have resulted from protocol requirements for supportive care and dosage modification according to hemoglobin level in the EMBRACA trial, which may not reflect routine clinical practice in the real-world setting.^13^

Findings from the real-world US study were also similar to those reported in the phase IV ViTAL study in France, which included 86 patients with g*BRCA*m HER2- LA/mBC, as the median TTF of talazoparib for US patients was 8.5 months and the median time to treatment discontinuation, defined as the time between the date of the first dose of talazoparib and the date of last dose or death, for patients in the French study was 8.6 months.^14^ No difference was observed according to HR status for either study. However, the median rwPFS and rwORR for talazoparib were more favorable in this real-world US study versus those observed in small real-world studies conducted in Turkey (*n* = 47) and Russia (*n* = 24). It should also be noted that patients in the Turkish and Russian studies were heavily pretreated and initiated talazoparib in later lines of therapy (3 or more lines of therapy were received prior to talazoparib among 10.7%, 51.5%, and 33.5% in the real-world studies set in the USA, Turkey, and Russia, respectively).^15,16^ The median rwPFS for talazoparib was 8.7 months (median follow-up period of 8.2 months) for the US study population and 6.5 months (median follow-up time of 13.6 months) for the Turkish *BRCA*-mutated breast cancer patient study population.^15^ In addition, rwORR for talazoparib was 63.1% for the US study population versus 31.9% for the Turkish study population.^15^ In the Russian study, the median rwPFS and rwORR for talazoparib were 6.5 months and 29.0% for patients with g*BRCA*-mutated HER2- metastatic breast cancer.^16^ It is likely that distinctions in patient characteristics and the small sample sizes of the Turkish and Russian studies may have impacted these differences, and rwPFS and rwORR were improved for subgroups treated with talazoparib in earlier lines.^15,16^

### Limitations

This study may be limited by unobserved data and missing data bias (eg, undercounting of events that are unknown to the abstracting oncologists because of having occurred outside the office/clinical setting, loss to follow-up if patients transferred care to other providers or clinics). Source document verification was not conducted; however, all physicians had been required to submit to data validation checks, and failure to correctly validate data resulted in exclusion. Moreover, this study included a limited number of patients (84), with data abstracted by a limited number of oncologists (9). Patients were selected based on prespecified selection criteria and, hence, findings may not be representative of all patients who have received talazoparib. In addition, treatment and testing patterns may not reflect those of all oncologists managing patients with LA/mBC. Although the study sample size was limited, the potentially eligible patient population is considered to be rare, given the fact that g*BRCA* mutations are detected in less than 5% of unselected patients with mBC,^6^ and g*BRCA* mutation testing is not universally performed. A US chart review study of 407 HER2-negative mBC patients reported that 47% were tested for *gBRCA1/2* mutation with or without somatic mutation. Testing also differed by patient age (more younger patients were tested), family history of *BRCA1/2*-related cancer (more patients with family history were tested), cancer subtype (more patients with TNBC versus HR-positive disease were tested), and care setting (patients were more likely to be tested in academic versus community practices).^17^ In terms of data related to supportive care medication use during talazoparib treatment, data collection did not distinguish whether patients were prescribed medication as prophylactic therapy or as needed. Finally, the findings of this study may be impacted by a lack of uniform assessment criteria for certain variables such as disease response.

## Conclusion

This study provides the first published findings from real-world g*BRCA*m HER2-LA/mBC patients treated with talazoparib outside the clinical trial setting. Importantly, the results are concordant with those of the EMBRACA trial, and randomized controlled trials remain the gold standard for the evaluation of safety and efficacy of pharmaceutical interventions. However, clinical outcomes in clinical trials and those observed in real-world populations may differ.^18^ Moreover, the main limitation of clinical trial data remains the lack of representativeness and generalizability of the sample population to real-world patients for whom the drug approval was indicated.^19,20^ In addition, barriers to clinical trial participation encompass sociocultural norms, health system impediments, and clinical attitudes and practices differentiating patient care by race and/or ethnicity.^20^ Due to the voluntary nature of participation in clinical trials, most US patients (approximately 95%) are not represented and may therefore experience outcomes not replicated in the real-world setting (eg, community or academic practice).^20,21^ This study included a racially/ethnically diverse cohort of patients similar in racial and ethnic distribution to that of the broader US population and was specific to real-world practice.

In conclusion, findings from this study, which represents the first such published evaluation conducted in the USA, show the clinical benefits of talazoparib treatment in g*BRCA*m HER2-LA/mBC in real-world practice. Clinical outcomes in this real-world population of patients treated with talazoparib were consistent with those reported in the EMBRACA randomized clinical trial.

## Supplementary Material

oyad021_suppl_Supplementary_Figure_S1

oyad021_suppl_Supplementary_Table_S1

## Data Availability

The data underlying this article cannot be shared publicly to maintain the privacy of individuals that participated in the study.
