# Supplementary material for: Characteristics, Treatment, and Outcomes of Real-World Talazoparib-Treated Patients With Germline BRCA-Mutated Advanced HER2-Negative Breast Cancer
Source: Oncologist. 2023 Mar 23;28(5):414–24. doi: 10.1093/oncolo/oyad021 (PMC10166159; doi:10.1093/oncolo/oyad021)
Supplement: oyad021_suppl_Supplementary_Figure_S1 [file oyad021_suppl_supplementary_figure_s1.docx]

**Supplemental Figure S1.** Sankey Diagram of Treatment Sequences


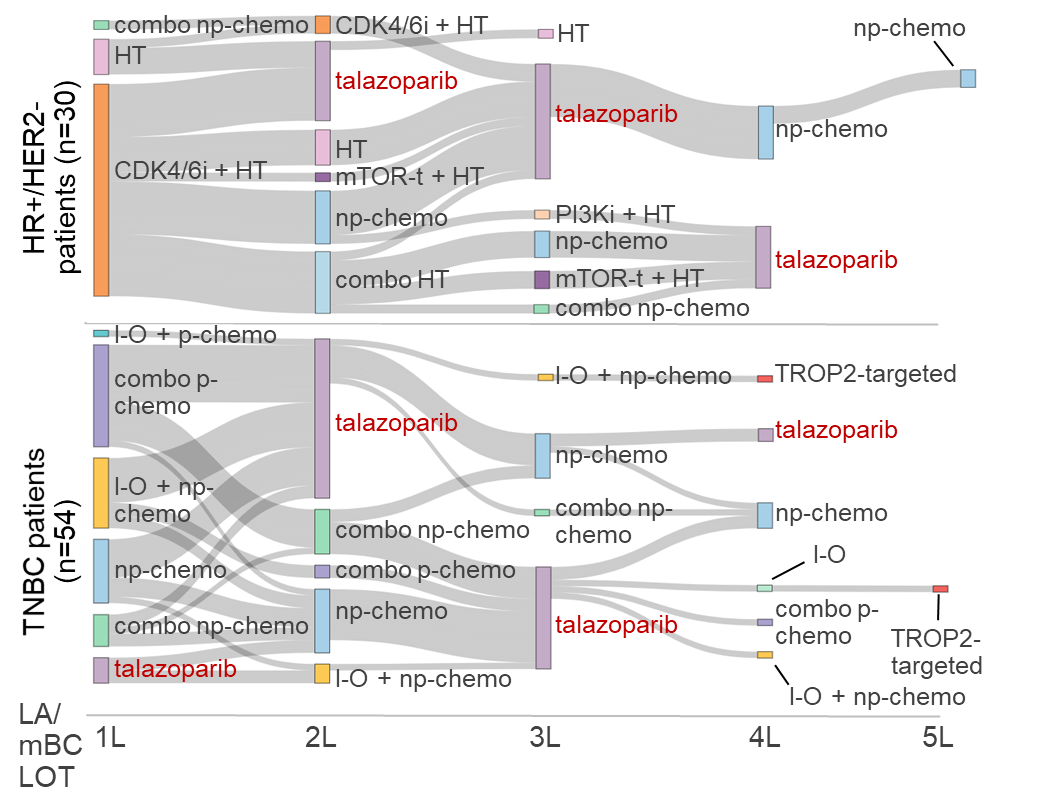


Sankey plots, depicting the sequence of regimen types received, with increasing lines of therapy from left to right (1L-5L). Distinct regimen types are denoted by different colors and the thickness of the links between nodes reflect the relative proportions of patients who received a given treatment sequence. Abbreviations: 1L, first-line, 2L, second-line; 3L, third-line; 4L, fourth-line; 5L, fifth line; CDK4/6i, cyclin dependent kinase 4/6 inhibitor; combo, combination; gBRCAm, germline breast cancer susceptibility gene mutated; HER2, human epidermal growth factor receptor; HER2-, human epidermal growth factor receptor 2 negative; HR-positive, hormone receptor positive; HT, hormonal therapy; I-O, mmune-oncology therapy; LA/mBC, locally advanced or metastatic breast cancer; LOT, line of therapy; mTOR-t, mammalian target of rapamycin-targeted therapy; np-chemo, non-platinum-based chemotherapy; p-chemo, platinum-based chemotherapy; PI3Ki, phosphatidylinositol-3-kinase inhibitor, TNBC, triple-negative breast cancer; TROP2, trophoblast cell surface antigen 2.
