# Supplementary material for: Characteristics, Treatment, and Outcomes of Real-World Talazoparib-Treated Patients With Germline BRCA-Mutated Advanced HER2-Negative Breast Cancer
Source: Oncologist. 2023 Mar 23;28(5):414–24. doi: 10.1093/oncolo/oyad021 (PMC10166159; doi:10.1093/oncolo/oyad021)
Supplement: oyad021_suppl_Supplementary_Table_S1 [file oyad021_suppl_supplementary_table_s1.docx]

**Supplementary Table S1.** Post-Talazoparib Treatment Patterns

|  | **All patients (N=84)** | **HR+ (n=30)** | **TNBC (n=54)** |
| --- | --- | --- | --- |
| Systemic therapy post-talazoparib, n (%) | 23 (27.4) | 7 (23.3) | 16 (29.6) |
| Number of lines of treatment post-talazoparib, n (%) |  |  |  |
| 1 | 17 (20.2) | 5 (16.7) | 12 (22.2) |
| 2 | 6 (7.1) | 2 (6.7) | 4 (7.4) |
| Treatment regimens received as any line of therapy post-talazoparib, n (% among patients with subsequent treatment)^a^ |  |  |  |
| Single-agent HT | 1 (4.3) | 1 (14.3) | 0 (0.0) |
| Single-agent CT (NPt-based) | 16 (69.6) | 6 (85.7) | 10 (62.5) |
| Combo CT (Pt-based) | 1 (4.3) | 0 (0.0) | 1 (6.3) |
| Combo CT (NPt-based) | 1 (4.3) | 0 (0.0) | 1 (6.3) |
| Single-agent TROP2-targeted therapy | 2 (8.7) | 0 (0.0) | 2 (12.5) |
| Single-agent I-O | 1 (4.3) | 0 (0.0) | 1 (6.3) |
| Combo I-O + CT (NPt-based) | 4 (17.4) | 0 (0.0) | 4 (25.0) |

^a^Not mutually exclusive.

Abbreviations: combo, combination; CT, chemotherapy; HER2, human epidermal growth factor receptor; HER2-, human epidermal growth factor receptor 2 negative; HR+, hormone receptor positive; HT, hormonal therapy; I-O, immuno-oncology therapy; LA/mBC, locally advanced or metastatic breast cancer; NPt, non-platinum; PI3Ki, phosphatidylinositol-3-kinase inhibitor; Pt, platinum; TNBC, triple-negative breast cancer; TROP2, trophoblast cell surface antigen 2.
